# A tandem CCCH type zinc finger protein gene *CpC3H3* from *Chimonanthus praecox* promotes flowering and enhances drought tolerance in *Arabidopsis*

**DOI:** 10.1186/s12870-022-03877-2

**Published:** 2022-10-29

**Authors:** Huamin Liu, Shiqi Xiao, Shunzhao Sui, Renwei Huang, Xia Wang, Huafeng Wu, Xia Liu

**Affiliations:** 1 College of Landscape Architecture and Life Science/Institute of Special Plants, Chongqing University of Arts and Sciences, Yongchuan, Chongqing, 402160 China; 2 Chongqing Engineering Research Center for Floriculture, Key Laboratory of Horticulture Science for Southern Mountainous Regions of Ministry of Education, College of Horticulture and Landscape Architecture, Southwest University, Chongqing, 400715 China; 3 College of Chemistry and Life Sciences, Sichuan Provincial Key Laboratory for Development and Utilization of Characteristic Horticultural Biological Resources, Chengdu Normal University, Chengdu, 611130 China

**Keywords:** CCCH type zinc finger protein, *CpC3H3*, Flowering, Drought tolerance, Wintersweet

## Abstract

**Background:**

CCCH-type zinc finger proteins play important roles in plant development and biotic/abiotic stress responses. Wintersweet (*Chimonanthus praecox*) is a popular ornamental plant with strong resistance to various stresses, which is a good material for exploring gene resource for stress response. In this study, we isolated a CCCH type zinc finger protein gene *CpC3H3* (MZ964860) from flower of wintersweet and performed functional analysis with a purpose of identifying gene resource for floral transition and stress tolerance.

**Results:**

*CpC3H3* was predicted a CCCH type zinc finger protein gene encoding a protein containing 446 amino acids with five conserved C-X_8_-C-X_5_-C-X_3_-H motifs. CpC3H3 was localized in the cell membrane but with a nuclear export signal at the N-terminal. Transcripts of *CpC3H3* were significantly accumulated in flower buds at floral meristem formation stage, and were induced by polyethylene glycol. Overexpression of *CpC3H3* promoted flowering, and enhanced drought tolerance in transgenic *A. thaliana*. *CpC3H3* overexpression affects the expression level of genes involved in flower inducement and stress responses. Further comparative studies on physiological indices showed the contents of proline and soluble sugar, activity of peroxidase and the rates of electrolyte leakage were significantly increased and the content of malondialdehyde and osmotic potential was significantly reduced in transgenic *A. thaliana* under PEG stress.

**Conclusion:**

Overall, *CpC3H3* plays a role in flowering inducement and drought tolerance in transgenic *A. thaliana*. The *CpC3H3* gene has the potential to be used to promote flowering and enhance drought tolerance in plants.

**Supplementary Information:**

The online version contains supplementary material available at 10.1186/s12870-022-03877-2.

## Background

Zinc finger protein is a superfamily with five types (C2H2, C3H, C3HC4, C2HC5 and C3H2C3) of proteins. Typical CCCH type zinc finger protein was defined as the proteins contain 1-6 copy C-X_6–14_-C-X_4–5_-C-X_3_-H type zinc finger motifs. CCCH type zinc finger protein gene was widely exist in plant species, studies have revealed that there are 68 C3H proteins in *A. thaliana*, 67 in rice, 68 in *Zea mays*, 34 in *Medicago truncatula* and 91 in *Populus trichocarpa*. Functional characterizations showed CCCH type zinc finger proteins play roles in various progresses of plant growth and stress responses.

The transition of vegetative growth to flowering is a key developmental progress in flowering plants which is important not only for environmental adaptation, but also for agricultural productivity. Studies on molecular mechanism and genetic basis of the transition in the model plant *A. thaliana* found that the transition is regulated by elaborate genetic pathways, namely the photoperiod, vernalization, gibberellic acid (GA), age, autonomous, and ambient temperature signaling pathways, in response to endogenous (age, GA), and environmental (day length, temperature, ambient temperature and stress) stimuli [1, 2]. More than 180 genes participate in floral transition [2], such as genes from various pathways, *CONSTANS 1* from photoperiod pathway, *SHORT VEGETATIVE PHASE* from ambient temperature pathway, *VERNALIZATION INSENSITIVE* (*VINs*) from vernalization pathway, *The GIBBERELLIN 20 OXIDASE* (*GA20ox*) from gibberellic acid pathway, and signal integrators, *FLOWERING LOCUS T* (*FT*), *SUPPRESSOR OF OVEREXPRESSION OF CONSTANS 1* (*SOC1*), *AGAMOUSLIKE 24* (*AGL24*), and floral meristem identity genes, *APETALA1* (*AP1*), *APETALA2* (*AP2*), *LEAFY* (*LFY*) [3–5]. CCCH type zinc finger proteins were also found play roles in floral transition, *MsZFN* (*Medicago sativa zinc finger protein*) a gene induced by dark from alfalfa delayed flowering in *A. thaliana* [6], and *AtC3H17* promotes flowering in *A. thaliana* [7], *Ehd4 (Early heading date 4)* acts as a critical regulator promoting flowing in rice in photoperiod pathway [8].

As one of the most severe natural stresses, drought affects the productivity and quality of plants [9]. Plants have evolved multifaceted adaptation strategies to recognize and adapt the drought stress at molecular, biochemical, physiological and morphological levels [10]. Numerous stress-induced genes are involved in the recognition and adaption of drought stress. According to the functional annotation, these genes can be divided into two groups, the first class involves in drought response through signal transduction, including the protein kinase genes (MAP kinase, CDB kinase), transcription factors (MYB, NAC, MYC and DREB). The second class involves in drought tolerance as function genes, such as late embryogenesis abundant (LEA) proteins, osmotin proteins, water channel proteins, stomatal movement proteins, sugar and proline transporters, oxidative enzyme, and various proteases [11]. CCCH type zinc finger protein genes were also found play roles in stress response, such as *OsC3H10*, *OsTZF5* (*Oryza sativa CCCH-tandem zinc finger protein 5*) improve drought tolerance in rice [12, 13], *AtSZF1* (*Arabidopsis thaliana salt-inducible zinc finger 1*), *AtSZF2* (*Arabidopsis thaliana salt-inducible zinc finger 2*), *GhZFP1* (*Gossypium hirsutum putative CCCH-type zinc finger transcription factor*) play role in salt stress response [14, 15].

Few CCCH type zinc finger protein genes have multiple functions in stress response and floral transition. Overexpression of *AtZFP1* (*Arabidopsis thaliana Zinc finger protein 1*) delays floral transition and enhances salt tolerance in *A. thaliana* [16], and overexpression of *AtTZF1* (*Arabidopsis thaliana tandem zinc finger protein 1*) delays flowering and enhanced cold and drought tolerance [17].

Wintersweet (*Chimonanthus praecox*) is a popular Chinese endemic shrub with bright yellow flowers and attractive fragrance. It blossoms in hard winter and shows strong stress resistance to drought, cold, heat and waterlogging [18, 19]. It is a good material for exploring stress response genes, but fewer genes from wintersweet were characterized [19–22]. We obtained a novel tandem CCCH type zinc finger protein gene *CpC3H3* (accession number: MZ964860) from flower of wintersweet previously, but is function was still unknown. We aimed to characterize the function of *CpC3H3* for identifying stress resistance gene resource.

## Results

### CpC3H3 isolation and sequence analysis

**Fig. 1:**
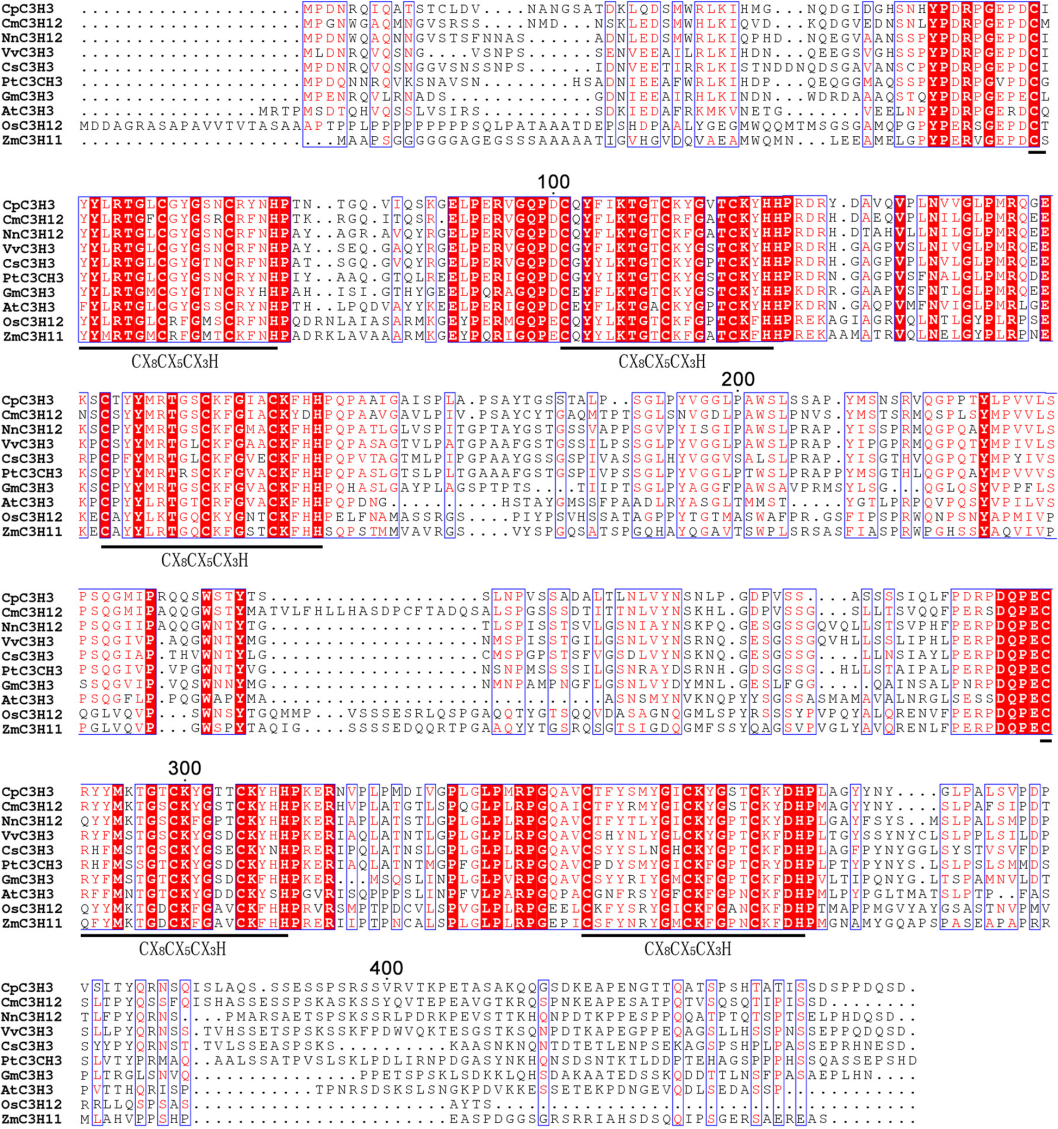
Multiple sequence alignment of CpC3H3 and its homologues from *A. thaliana, Zea mays, Populus trichocarpa*, *Cinnamomum micranthum*, *Nelumbo nucifera*, *Vitis vinifera*, *Glycine max*, and *Camellia sinensis.* The blank lines indicate the position of the C-X_8_-C-X_5_-C-X_3_-H type motifs; Red box, white character indicates the strict identity of the residues, red character indicates the similarity residues in a group and the blue frame indicates the similarity residues across groups

The *CpC3H3* CDS contained 1341 bp that encoded a 446 amino-acid protein with a calculated molecular mass of 48.43 KDa. Sequence analysis showed that five putative conserved C-X_8_-C-X_5_-C-X_3_-H motifs were detected in CpC3H3 (Fig. 1). Multiple sequence alignments performed among CpC3H3 and its homologues from model plants (*Arabidopsis thaliana*, *Oryza sativa*, *Populus trichocarpa*, etc) and other species (*Cinnamomum micranthum*, *Nelumbo nucifera*, etc.) showed that they were highly conserved and shared the same numbers and types of the CCCH motifs which were important for their functions (Fig. 1).

**Fig. 2:**
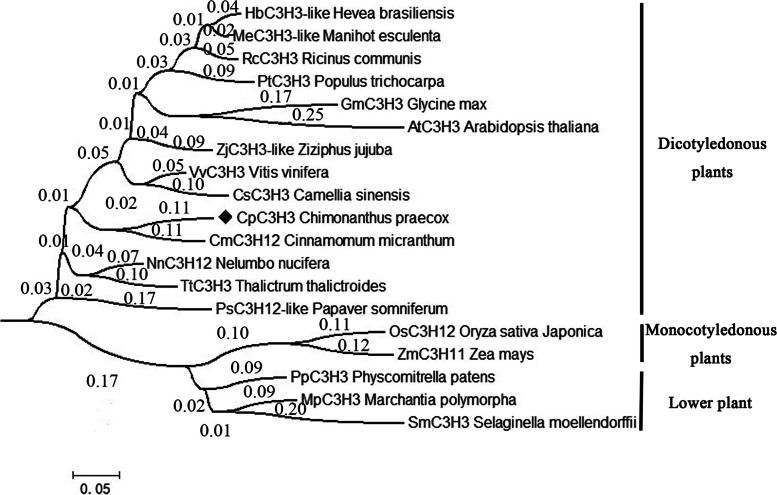
Phylogenetic analysis of CpC3H3 and its homologues from lower plants *Selaginella moellendorffii*, *Physcomitrella patens*, *Marchantia polymorpha*, Monocotyledonous plants *Oryza sativa*, *Zea mays*, and Dicotyledonous plants *Arabidopsis thaliana, Populus trichocarpa*, *Cinnamomum micranthum*, *Nelumbo nucifera*, *Vitis vinifera*, *Glycine max*, *Camellia sinensis*, *Ricinus comunis*, *Hevea brasiliensis*, *Manihot esculenta*, *Thalictrum thalictroides*, *Ziziphus jujuba*, and *Papacer somniferum*

The phylogenetic analysis of CpC3H3 and other homologues from various plants showed that the C3H3 proteins were divided into two main branches, proteins from lower plants like *Selaginella moelledndorffii*, *Marchantia polymorpha*, and *Physcomitrella patents* and Monocotyledonous plant, such as *Zea mays*, *Oryza sativa* were clustered within one clade, and the Dicotyledonous plant C3H3 proteins were in the other branch where CpC3H3 was belonged to (Fig. 2). This finding indicated that the C3H3 proteins of Dicotyledonous plants may share some distance from that of Monocotyledonous and lower plants which may ultimately lead to the function difference among these plants.

*Cis*-acting elements prediction revealed that 12 hormone responsive elements, including 2 ABRE, 4 MYC ABA responsive elements, 4 MeJA responsive elements, 1 Ethylene responsive elements and 1 Auxin responsive elements, and 10 stress responsive elements, including 2 TC-rich repeats (*Cis*-acting element involved in defense and stress responsiveness), 1 MBS (myb binding site involved in drought-inducibility), 4 MYB binding site, 1MYBHv1 biding site and 2 ARE (Anaerobic induction) elements and 16 light responsive *cis*-elements were predicted in the 2138 bp upstream sequence of *CpC3H3* (supplementary table S2).

### Subcellular localization analysis

**Fig. 3:**
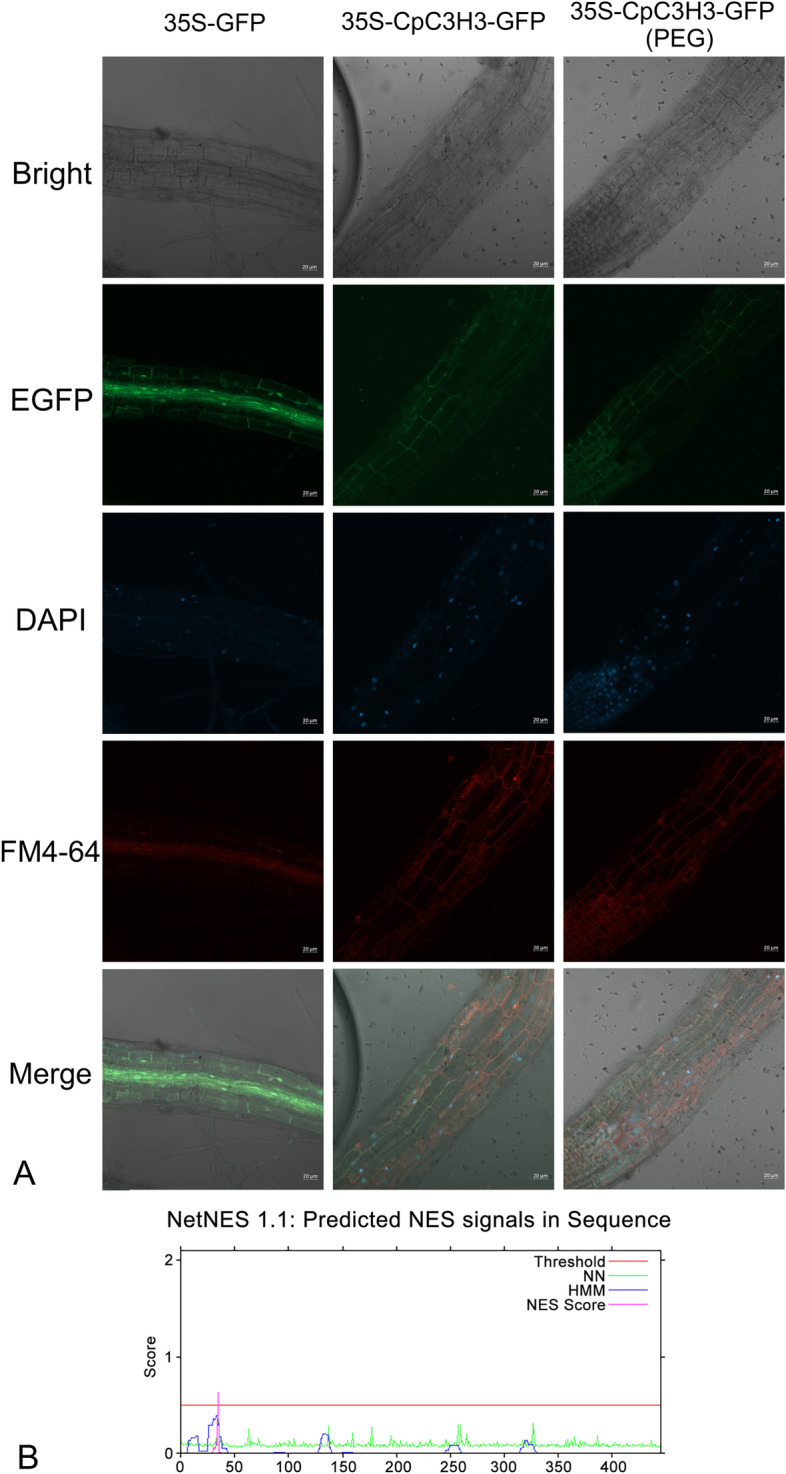
Subcellular localization analysis of the CpC3H3. **A** Subcellular localization of the CpC3H3 protein in root of transgenic *A. thaliana*. 35S-GFP was served as the control. Fluorescence of 35S-GFP was detected throughout the cell while the 35S-CpC3H3-GFP was co-localized with RFP of FM 4-64 in the cell membrane. **B** Nuclear export signal (NES) was predicted by online tool of NetNES 1.1, putative NES sequence was found between amino acids 30 to 37. Bars = 20 μm

The subcellular localization of CpC3H3 was first predicted by online tool, result showed that CpC3H3 was predicted localizing to cell membrane. And to further examine the subcellular localization of CpC3H3, the roots of the transgenic *A. thaliana* plants carrying 35S:GFP and 35S:CpC3H3-GFP were used to detect the GFP signal respectively. GFP observation n revealed that the GFP signal generated by CpC3H3-GFP was observed in cell membrane which was co-localized with the RFP generated by membrane marker FM4-64 while the control 35S:GFP was in the nucleus and cytoplasm (Fig. 3A), this result indicated that CpC3H3 is mainly located in cell membrane. But Nuclear export signal (NES) prediction showed that there was a putative nuclear export signal (NES: between amino acids 30 to 37) at the N-terminal of CpC3H3 (Fig. 3B). Few other CCCH proteins with NES signal can shuttle from nucleus to other organelles under certain conditions. We assume CpC3H3 might be a shuttling protein under drought stress, so we also detected the subcellular localization of CpC3H3 after PEG treatment, but no significant difference of GFP signal was observed (Fig. 3A).

### Expression patterns of *CpC3H3* in *C. praecox*

**Fig. 4:**
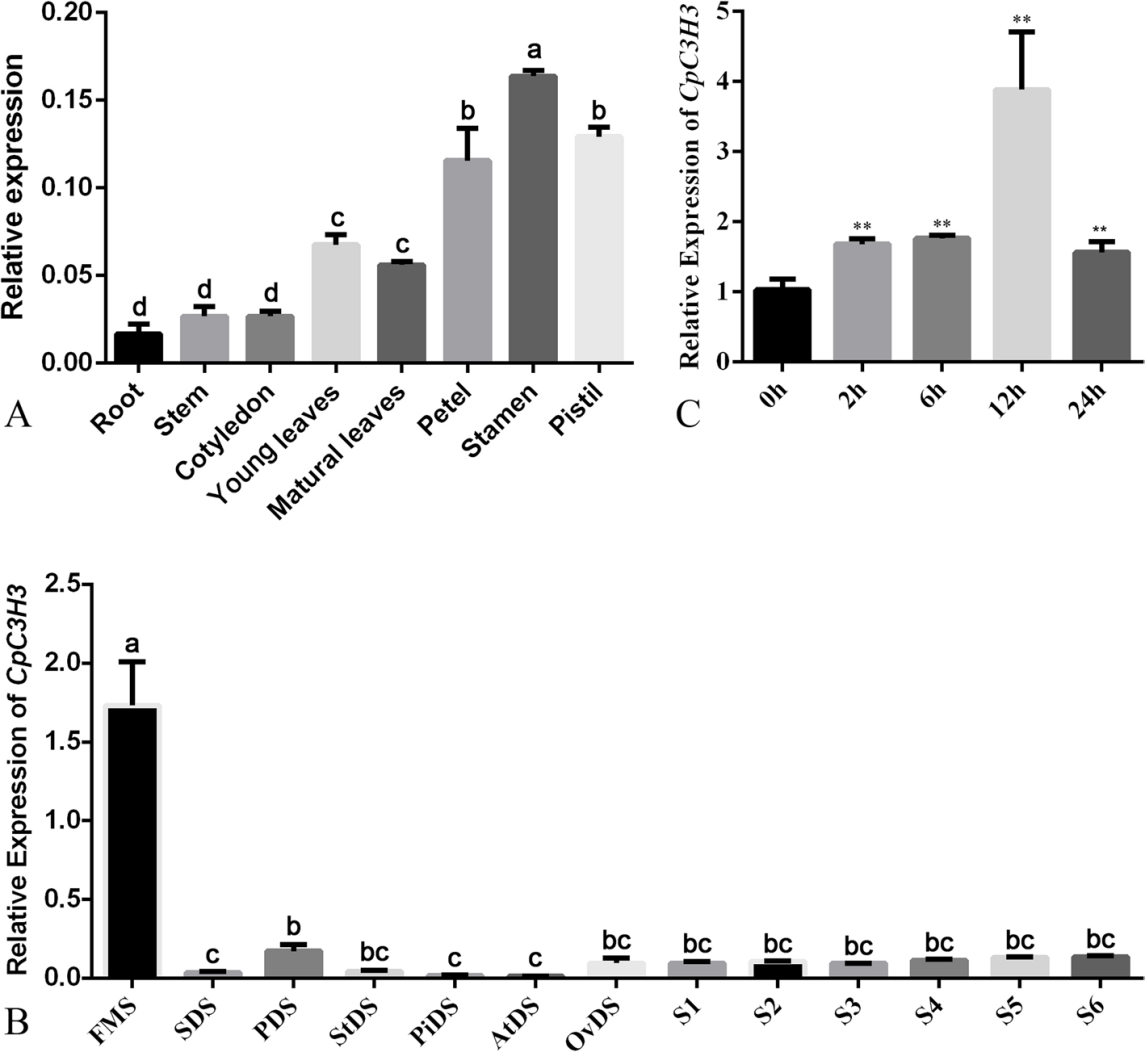
Expression pattern of *CpC3H3* in wintersweet. **A**, relative expression of *CpC3H3* in various tissues of wintersweet; **B**, expression pattern of *CpC3H3* in flower developmental stages; **C**, relative expression of *CpC3H3* in leaves in response to 20% PEG after 0 h, 2 h, 6 h, 12 h, 24 h. FMS, flower meristem formation stage; SDS, sepal primordium deferential stage; PDS, petal primordium deferential stage; StDS, sepal primordium deferential stage, PiDS, Pistil primordium deferential stage; AtDS, anther developmental stage; OvDS, ovule developmental stage; S1, flower bud stage; Stage 2, petal-display; Stage 3, initiating bloom; Stage 4, bloom; Stage 5, early-withering, and Stage 6, late-withering. The * and ** in fig. 4C indicate a significant difference from 0 h at *p* < 0.05 and *p* < 0.01, respectively, as determined by the Student t-test. Mean values followed by the same letter are not significantly different according to LSD multiple range test in fig. 4A and Games-Howell test in fig. 4B (*P* ≤ 0.01) respectively. The value of each bar represents mean ± SE (*n* = 3) of three replicates

Tissue specific expression analysis in cotyledons, roots, stems, young leaves, mature leaves, petals, stamens and pistils showed that *CpC3H3* was widely expressed in tissues of wintersweet, but its expression level is higher in flower organs than in vegetative organs (Fig. 4A). The dynamic expression of *CpC3H3* in flower at different developmental stages showed that *CpC3H3* was more abundantly expressed in floral meristem formation stages than in other stages (Fig. 4B). The results of induced expression analysis showed that *CpC3H3* was induced by PEG, and its expression reached a peak 12 hours after treatment with an expression level 4 times that of untreated. (Fig. 4C).

### Overexpressing of *CpC3H3* promotes flowering in *A. thaliana*

To investigate the functions of CpC3H3, the CDS of *CpC3H3* was introduced into *A. thaliana* under the control of the 35S promoter. Transgenic lines were confirmed by PCR and qRT-PCR using the DNA and cDNA as the template (supplementary Fig. S1-S2), and the line 10, 14 and 18 were then chosen for further phenotype observation and related gene expression analysis.

**Fig. 5:**
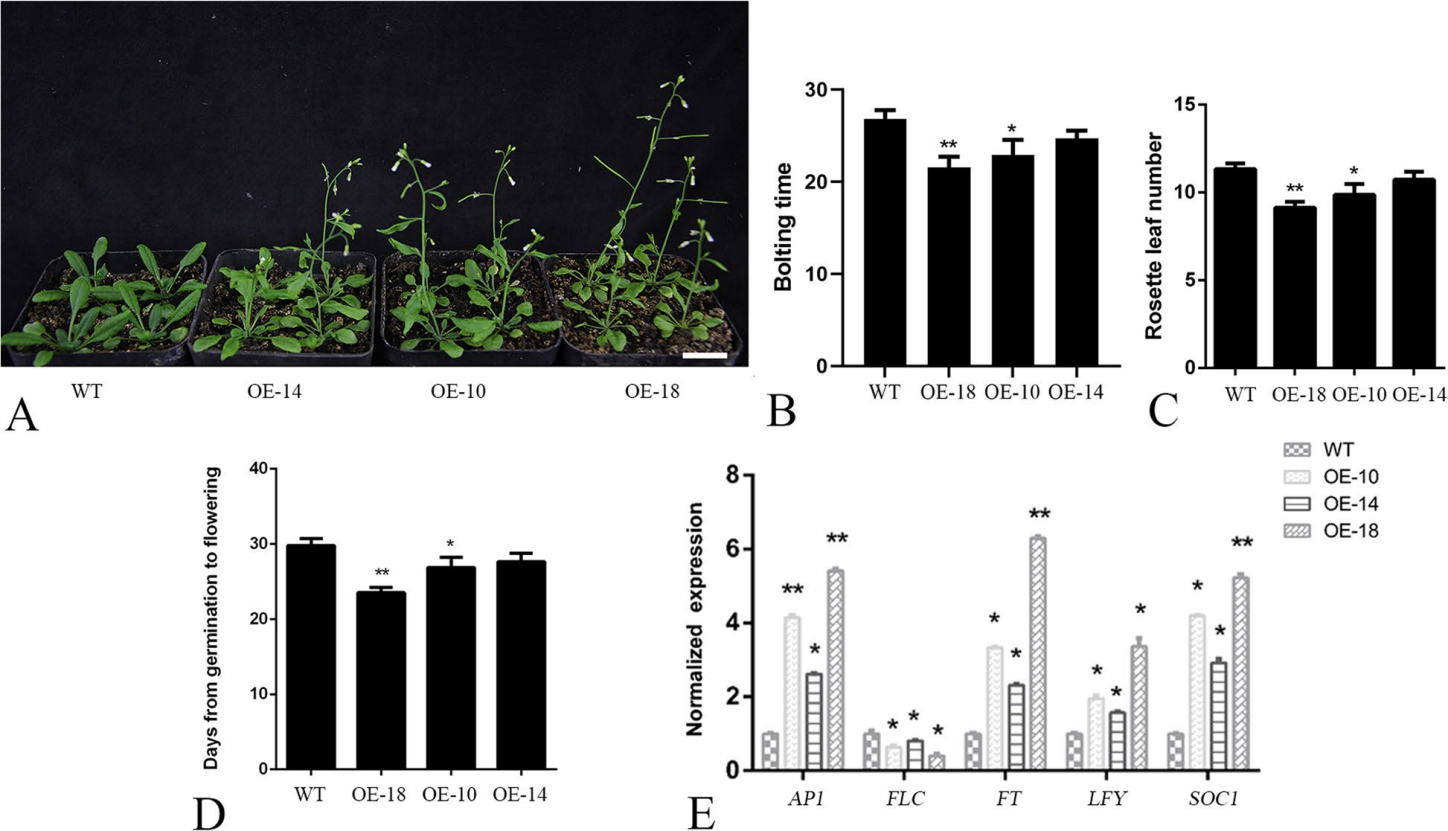
Overexpression of *CpC3H3* promote floral transition. WT, wild type; OE-18, overexpression line-18; OE-10, overexpression line-10; OE-14, overexpression line-14; the error bars represent the standard deviation per triplicate. The * and ** indicate a significant difference from WT at *p* < 0.05 and *p* < 0.01, respectively, as determined by the Student’ t-test. Bars = 2 cm

To observe the phenotype of the *CpC3H3* overexpression (OE) plants, the OE plants and WT plants were raised in a greenhouse under long day condition. We found that, the OE plants required an average of 23.7 days to bolting and 25.7 days from germination to flowering, while WT plants needed 27.1 days and 29.8 days respectively (Fig. 5A, B,D), and the rosette leaves of OE plants were less than that of WT (Fig. 5C). We also detected the expression levels of the key genes involved in flowering, results showed that the expression of the flowering promoter *AP1*, *FT*, *LFY* and *SOC1* were upregulated in transgenic plants while the flowering repressor *FLC* was downregulated (Fig. 5E). These results indicated that *CpC3H3* has a role in flowering inducement in *A. thaliana*.

### Overexpressing of *CpC3H3* enhances drought tolerance in *A. thaliana*

**Fig. 6:**
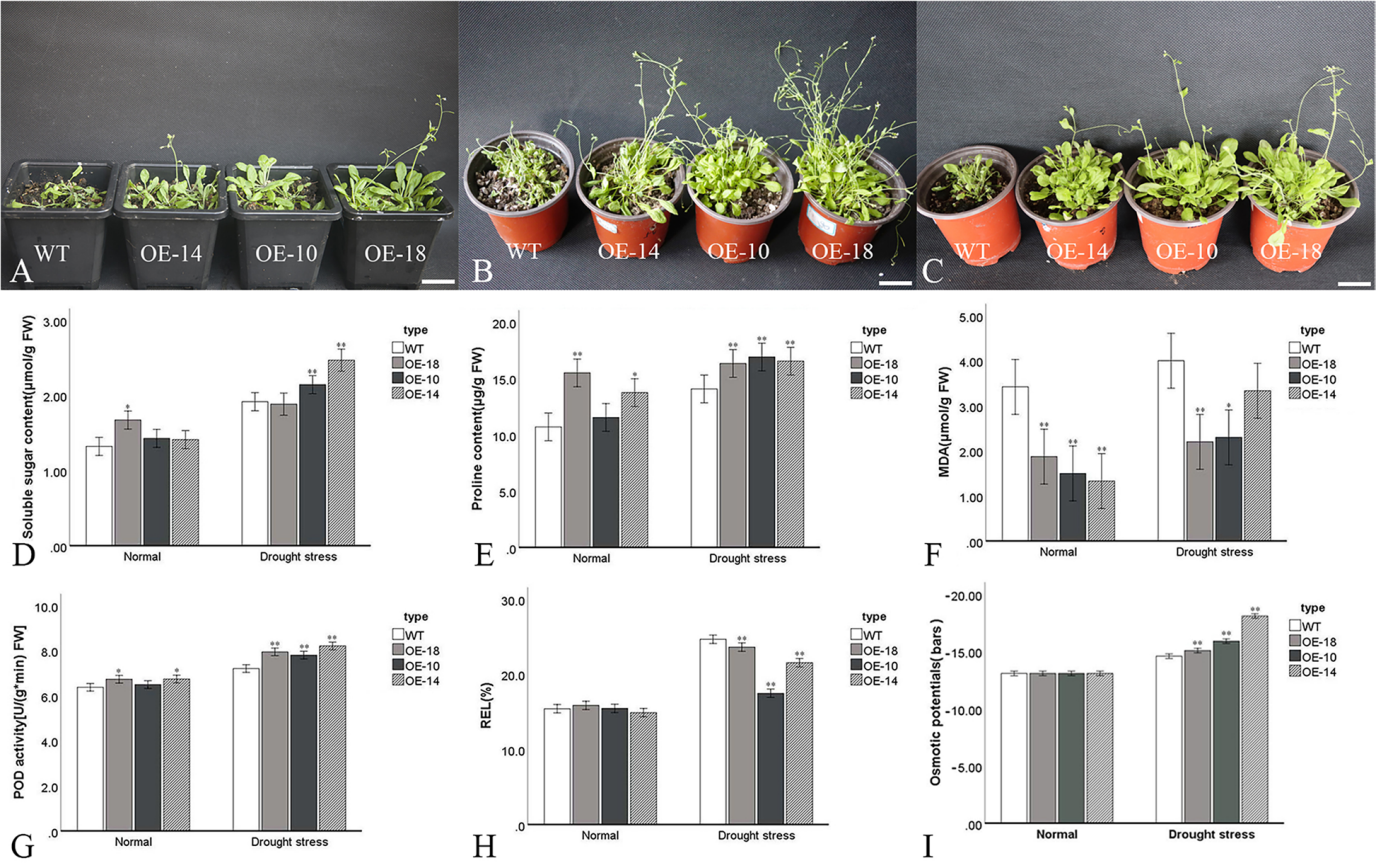
Overexpression of *CpC3H3* enhance drought tolerance. **A**-**C**, transgenic and WT *A. thaliana* plants treated with PEG, natural water deficit and mannitol; **D**-**G**, The content of soluble sugar, proline, MDA, activity of POD, electrolyte leakage rates and osmotic potentials in the transgenic *A. thaliana* plants and WT. The OE-plants and WT plants were treated with PEG after 24 h. OE-18, overexpression line-18; OE-10, overexpression line-10; OE-14, overexpression line-14. the error bars represent the standard deviation per triplicate. The * and ** indicate a significant difference from WT at *p* < 0.05 and *p* < 0.01, respectively, as determined by the student’ t-test. Bars = 2 cm

The expression of *CpC3H3* was induced by PEG, *CpC3H3* was deemed to play some roles in drought tolerance. Four-week-old WT and transgenic *A. thaliana* plants grown in soil were treated with 20% PEG6000, 150 mmol/L mannitol and planted without watering to evaluate the drought tolerance. The leaves of wild type plants were severely withered after the treatment, while that of overexpression lines were still robust (Fig. 6A-C).

Several physiological indices widely used to evaluate the plant stress response were determined in WT and transgenic plants under normal and drought condition. The content of soluble sugar showed little difference under normal condition but increased more significantly in overexpression strains than in wild type *A. thaliana* under drought stress (Fig. 6D), and the contents of proline were higher in transgenic plants under both normal and drought condition (Fig. 6E). As a marker for lipid peroxidation, content of MDA reflects the resistance ability of plants to stress, as shown in Fig. 6F, MDA content was higher in WT plants both in normal and drought conditions. As an important ROS scavenging antioxidant enzyme, activity of POD was significantly higher in the transgenic plants in both normal and drought conditions (Fig. 6G). The electrolyte leakage rates were almost the same in both transgenic and WT plants under normal condition, but were significantly lower in *CpC3H3*-overexpression *A. thaliana* plants after PEG stress treatments (Fig. 6H). The osmotic potentials showed no significant difference in WT and transgenic plants in normal condition, but significantly higher in transgenic plants after treated with PEG (Fig. 6I). The significant difference of proline, MDA and POD in normal condition, and the higher content of soluble sugar, proline, osmotic potential, activity of POD and lower REL and MDA under drought condition indicated that overexpression of *CpC3H3* enhanced the drought tolerance of transgenic *A. thaliana*.

### Identification of genes involved in the CpC3H3-regulated flowering and drought tolerance

To explore the regulatory mechanisms of *CpC3H3* mediated floral transition and drought tolerance, RNA sequencing was performed with twenty-day-old WT and OE plants. Genes with more than 2-fold differential expression levels in WT and overexpression lines are identified to be regulated by *CpC3H3* overexpression. 79 up- and 44 down-regulated genes were identified in the transgenic plants (Table S3). Since *CpC3H3* was mainly expressed in wintersweet flowers and induced by PEG6000, and its overexpressors exhibited early flowering and drought tolerance, we attempted to focus on the genes which were both regulated by *CpC3H3* overexpression and play roles in the floral transition or (and) stress tolerance. In terms of flowering, 15 genes, including the GATA transcription factors, bHLH transcription factors, etc., were annotated playing roles in floral transition according to Tair. 7 out of 15 were predicted components of the photoperiod pathway, and 8 of which were hormone related proteins (Table.1). And in the aspect of drought tolerance, 26 protein genes, including transcription factors (e.g.,MYB, APETALA2/ERF, NAC), oxidation-reduction process-related protein (e.g. cytochrome P450 family proteins), protein kinases (e.g.MAP 3 K), Dehydration-responsive protein DREB2A, compatible solute-related protein mannose-binding-lectin1 (MNB1), carbohydrate metabolism–related proteins UDP-glycosyltransferase, E3 ubiquitin-protein ligase ATDIP2 (DNA Binding Protein Interacting Protein 2), cell wall formation related protein pectin methylesterase inhibitor 11 and 13 (PMEI11, PMEI13), transport related protein genes (e.g. PHO1) and stomatal movement protein expansin1 (EXPA1), were annotated corresponding to drought tolerance (Table 1).

**Table 1:** Differential expression genes related to flowering and drought tolerance

	Gene	Annotation Flodchange		Gene	Annotation Flodchange	
Flowering	**Photoperiod**			**hormone related**		
	AT1G10470	ARR4	2.7	AT1G19050	ARR7	3.9
	AT2G18300	HBI1	2.3	AT3G48100	ARR5	3.5
	AT5G52390	PAR1	0.3	AT1G74890	ARR15	4.0
	AT5G56860	GATA21	2.0	AT1G74670	GASA6	5.7
	AT1G18400	BEE1	2.3	AT5G62920	ARR6	15.3
	AT1G18330	EPR1	2.5	AT3G57040	ARR9	2.4
	AT5G63470	AtNF-YC-4	2.2	AT3G63110	IPT3	5.3
	AT4G26150	CGA1	3.2			
Drought	**Signal transduction**			**Functional protein**		
	AT1G56650	MYB75	0.2	**osmotin**		
	AT1G57560	MYB50	0.4	AT1G68740	PHO1	2.2
	AT1G66390	MYB90	0.1	AT1G32900	GBSS1	0.2
	AT5G22380	NAC90	0.5	AT1G78830	MNB1	3.3
	AT5G44210	ERF9	2.0	**Cell protection**		
	AT3G16770	AtEBP	3.0	AT5G62350	PMEI11	2.5
	AT4G23750	CRF2	2.8	AT5G62360	PMEI13	2.7
	AT1G76040	CDBK 29	0.5	AT1G51270	vesicle-associated protein 1-4	0.3
	AT2G30040	MAP 3 K	3.1	**oxidation-reduction process-related proteins**		
	AT4G17660	PBS1-like protein 20	0.2	AT1G01190	CYP78A8	12.6
	AT5G05410	DREB2A	2.4	AT3G26200	CYP71B22	0.3
	AT2G18700	AtTPS11	2.3	AT5G52320	CYP96A4	0.5
	AT5G02580	Argininosuccinate lyase	0.3	AT1G33730	CYP76C2	0.2
	AT5G03210	AtDIP2	0.4	**stomatal movement**		
	AT2G41560	Calcium-transporting ATPase 4	5.2	AT1G69530	EXPA1	2.8

**Fig. 7:**
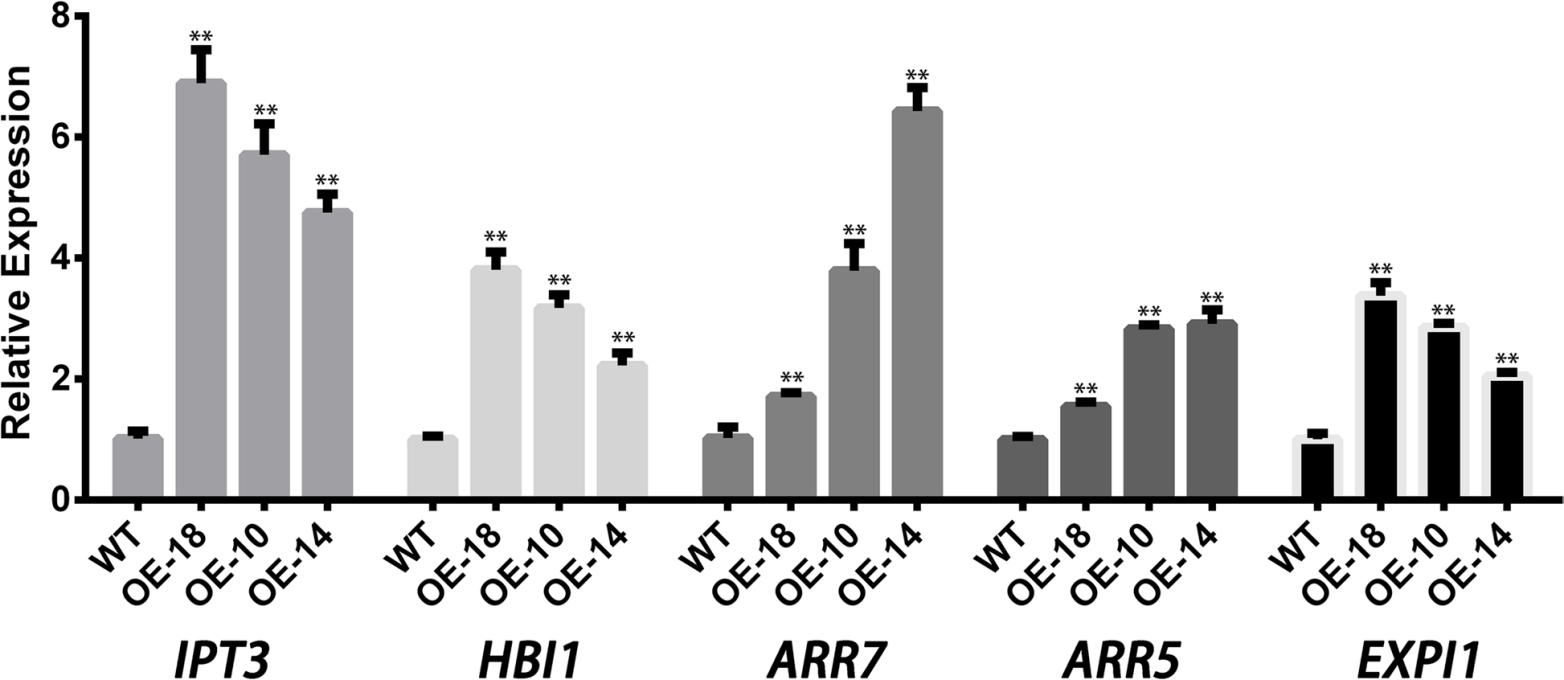
Expression validation of the genes randomly picked from the RNA-sequencing profile. WT, wild type; OE-18, overexpression line-18; OE-10, overexpression line-10; OE-14, overexpression line-14; the error bars represent the standard deviation per triplicate. The * and ** indicate a significant difference from WT at *p* < 0.05 and *p* < 0.01, respectively, as determined by the Student t-test

We further confirmed the expression patterns of several genes, *Isopentenyltransferase 3* (*IPT3*), *HOMOLOG of BEE2 INTERACTING WITH IBH 1* (*HBI1*), *Arabidopsis Thaliana Response Regulator 5* and *7* (*ARR5, ARR7*), and *Expansin-A1* (*EXPA1*), randomly picked from the candidates identified in WT and OE plants by qRT-PCR. The analysis showed their expression patterns were similar to the expression data derived from RNA sequencing analysis (Fig. 7).

## Discussion

### CpC3H3 is a tandem CCCH zinc finger protein

The C-X_8_-C-X_5_-C-X_3_-H type zinc finger motif was found in most of the CCCH type zinc finger proteins, 44 of 68 *A. thaliana* CCCH type zinc finger proteins, and 36 of 67 rice CCCH proteins contain the C-X_8_-C-X_5_-C-X_3_-H motif [23], and 82% of *Populus* CCCH proteins contain the conventional C-X_7_-_8_-C-X_5_-C-X_3_-H motifs [24]. The C-X_8_-C-X_5_-C-X_3_-H motif may be an ancient CCCH motif. Since CpC3H3 contains 5 tandem C-X_8_-C-X_5_-C-X_3_-H motifs, and results of multiple sequence alignment and phylogenetic analysis showed that CpC3H3 shared high conservation with its homologues, even the homologues from the lower plant *physcomitrella patent*, *Selaginella moelledndorffi, CpC3H3* might be an ancient gene with conserved functions.

### CpC3H3 might be a shuttling protein

CCCH-type zinc finger proteins can localize to different position of cells, some proteins can localize to nucleus with transcriptional activities [7, 15, 25], some can localize to plasma membrane [26], some can localize to cytoplasmic [13, 27], and some of them are shuttling proteins [12, 28–30]. In our study, subcellular localization analysis showed CpC3H3 was localized in plasma membrane, but NES sequence prediction showed CpC3H3 contained a NES sequence at the N-terminal which showed some similarities with other shuttling CCCH proteins. GhZFP1 which function in salt resistance was located in nuclear but with a NES peptide [15]; *AtTZF1* and *AtTZF9* (*Arabidopsis thaliana tandem zinc finger protein 9*) function in stress response can traffic between the nucleus and cytoplasm [31]. These implied that CpC3H3 might be a shuttling protein like other CCCH shuttling proteins, therefore, we detected the subcellular location of CpC3H3 after PEG treatment, but no significant difference was observed, this result showed CpC3H3 can’t shuttling under PEG stress. The subcellular localization property of CpC3H3 is still need to be further explored.

### *CpC3H3* promote floral transition in *A. thaliana*

The functions of CCCH proteins are closely related to their expression patterns [12, 13, 31], *AtC3H3* is preferentially expressed in vascular tissue, and highly expressed in the secondary wall forming tissues, has a function in cell wall elongation [32], *CpCZF1* and *CpCZF2* (*Chimonanthus praecox C3H-type zinc finger protein gene 1 and 2*), which was expressed highly in stamen primordium differentiation stage, affect stamen development [25]. In this study, tissue specific expression analysis revealed that *CpC3H3* was highly expressed in the flower organs, and further temporal expression analysis also showed that *CpC3H3* was mainly expressed in floral meristem formation stage rather than other flower development stages (Fig. 4). The expression patterns indicate that *CpC3H3* could play some roles in floral transition or development. Further functional characterization performed by overexpressing *CpC3H3* in *A. thaliana* showed that the OE lines bolted and flowered earlier and had less rosette leaves, showed upregulated flower promoters and downregulated flower repressor (Fig. 5C), these results implied that overexpression of *CpC3H3* promote flowering in *A. thaliana*. This is highly consistent with the former expression analysis, and shares some similarities with other CCCH-type zinc finger proteins. *MsZFN* from alfalfa delays flowering in *A. thaliana* with its transcripts increased under continuous dark conditions [6]. Ehd4, a CCCH type zinc finger protein from rice, showed a diurnal expression pattern which accumulates after dusk and reaching a peak at dawn, and then damping rapidly, regulate flowering in photoperiod pathway [8]. All in all, *CpC3H3* which expressed highly in flower of floral meristem formation stage played a role in flowering transition.

### *CpC3H3* may promote flowering through photoperiod and hormone signal pathways

To explore the regulation pathway that *CpC3H3* regulate flowering, transcriptome profile in WT plants and OE plants were analyzed. The transcriptome analysis revealed among the 123 DEGs, 15 genes were functionally associated with flowering (Table 1). Some genes are participating in flowering in Photoperiod pathway, such as *Early Phytochrome Responsive1* (*EPR1*), *Brassinosteroid Enhanced Expression1* (*BEE1*), *Arabidopsis Thaliana Response Regulator 4* (*ARR4*), *HBI1*. *EPR1* is regulated by phytochrom A and phytochrom B, and its overexpressors delayed flowering in *A. thaliana* [33]; *BEE1* is a positive regulator of photoperiod flowering, promote flowering by directly binding to the floral integrator FT [34]; *ARR4* is critical for proper circadian period [10]; HBI1, a basic helix-loop-helix protein, was regulated by light and affected flowering when overexpressed in *A. thaliana* [35]; Nuclear factor YC protein 4 (NFYC4) is required for CONSTANS-mediated, photoperiod-dependent flowering in *A. thaliana* [36]. And others, such as *IPT3*, *ARRs* and *GA-STIMULATED ARABIDOPSIS 6* (*GASA6*), are involved in flowering in hormone signal pathways. Cytokinin (CK) play role in flowering by activating the Twin Sister of FT (TSF) [37]. CK biosynthesis gene *IPT3* and receptor gene *ARR5*, *ARR6*, *ARR7*, *ARR9*, *ARR15* were upregulated in overexpression lines. *GASA6* is a GA-inducible and ABA-repressible gene which accelerated flowering when overexpressed in *A. thaliana* [38]. Flowering regulation mechanism of these DEGs implied *CpC3H3* may promote flowering through Photoperiod and hormone signal pathways, but further verification is needed.

### C*pC3H3* enhances drought tolerance in *A. thaliana*

*Cis*-acting elements such as ABRE, ERE, MBS and TC-rich repeats are commonly associate with stress response [20, 39], their occurring in the promoter regions implied that *CpC3H3* may have some role in stress response. Expression analysis figured out *CpC3H3* was induced by PEG, and functional analysis performed by overexpression *CpC3H3* in *A. thaliana* also showed that the OE plants are more tolerance than the WT plants when treated with PEG, this was similar to a lot of drought response genes, such as *IbZFP1*, *RICE CENTRORADIALIS 1* (*RCN1*), *RhEXPA4* [40–42]. Content of osmolytes, lipid peroxidation, ROS scavenging ability, electrolyte leakage rates are important reflections of plant stress resistance. Soluble sugar and proline are important osmolytes, osmotic potential could directly reflect the ability of plants to resist drought, MDA is a maker for lipid peroxidation, POD is one of an important ROS scavenging antioxidant enzyme, and electrolyte leakage rate is a reflection of cell death. These physiological indices have been widely used to evaluate the plant stress response. Contents of proline, MDA, activity of POD were significantly different under normal condition which indicated overexpression of *CpC3H3* affected these indices and thus enhanced drought tolerance. Further studies also showed contents of proline, soluble sugar and activity of POD were higher, and the content of MDA, osmotic potential and REL was lower in transgenic plants under drought condition. We also tested the osmotic potential of leaves of wintersweet under normal and PEG treatment, the osmotic potential was − 19.5 bars in normal condition, but decreased to − 22 bars after PEG treatment, which showed the same trend with the osmotic potential in transgenic plants. These results implied *CpC3H3* plays role in drought tolerance.

### *CpC3H3* may enhance drought tolerance by regulating the stress responsive genes

In response to drought stress, plants tend to alleviate the drought stress by activating the expression of the regulative and functional genes involved in shutting up stoma, developing roots, osmotic regulation, scavenging of reactive oxygen species, etc. In this study, 26 out of 123 differential expression genes are annotated stress-associated. 15 genes, including *MYBs*, *NAC DOMAIN CONTAINING PROTEIN 90* (*NAC90*), *ERF DOMAIN PROTEIN 9* (*ERF9*), *ETHYLENE-RESPONSIVE ELEMENT BINDING PROTEIN* (*AtEBP*), *CYTOKININ RESPONSE FACTOR 2* (*CRF2*), *CALCIUM-DEPENDENT PROTEIN KINASE 29* (*CDBK 29*), *MITOGEN-ACTIVATED PROTEIN KINASE KINASE KINASE 14* (*MAP 3 K*), *PBS1-LIKE PROTEIN 20*, *DEHYDRATION-RESPONSIVE ELEMENT BINDING PROTEIN 2* (*DREB2A*), *TREHALOSE-6-PHOSPHATE SYNTHASE 11* (*AtTPS11*), Calcium-transporting ATPase 4, argininosuccinate lyase, *DBP-INTERACTING PROTEIN 2* (*AtDIP2*), were function in stress-related signal transduction [43–45]. *PHO1*, *MNB1*, and *GRANULE BOUND STARCH SYNTHASE 1* (*GBSS1*) were function in osmotic equilibrium [46, 47]. *PMEI11*, *PMEI13*, *AtDIP2* play roles in plant cell protection [48, 49]. And the four CYPs were involved in membrane lipid antioxidation. *AtEXPA1* is a stomatal opening rate controller of *A. thaliana* [50]. Functional annotation of these genes indicates that overexpression of C*pC3H3* may enhance drought tolerance through regulating the stoma closing, osmotic balance, ROS scavenging and plant cell protecting. Additionally, the results of the determination of the physiological indices strengthened this conjecture.

## Conclusions

In conclusion, our results demonstrate that CpC3H3, a tandem CCCH type zinc finger protein with 5 C-X_8_-C-X_5_-C-X_3_-H motifs from wintersweet, was conserved with its homologues from other plants. Subcellular localization analysis showed CpC3H3 was located in cell membrane, but with a NES at the N-terminal which implied CpC3H3 might be a shuttling protein but could not shuttle under PEG treatment. *CpC3H3* was expressed highly in flower organs and floral meristem formation stage of wintersweet, and induced by PEG. Overexpression of *CpC3H3* caused early flowering and enhanced drought tolerance in *A. thaliana*. Transcriptome analysis revealed 15 and 26 of 123 DEGs were associated with flowering and stress response respectively. The content of MDA, proline, soluble sugar, osmotic potential, the activity of POD, and the rate of electronic leakage were significantly different in WT and transgenic *A. thaliana* under drought stress. All the results indicated that overexpression of *CpC3H3* promotes floral transition and enhances drought tolerance in *A. thaliana*. These findings not only extend our understanding of function of the CCCH-type zinc finger proteins, but also provide useful gene resource that can regulate flowering and drought tolerance. Moreover, *CpC3H3* is the first functional studied *C3H3s*, the characterization of *CpC3H3* also provide valuable reference for the study of C3H3 gene function in other species.

## Methods

### Plant materials and growth condition

Roots, stems, cotyledons, young leaves, mature leaves, flower organs (petal, stamen, pistil) were collected for detecting the expression level of *CpC3H3* in wintersweet. Seedlings of wintersweet were raised in the greenhouse with a relative humidity of 70%, a photoperiod of 16-h light (120 umol m^− 2^ s^− 1^, 25 °C) /8-h dark (20 °C) and the adult plants were planted in the campus of southwest university of China. Wintersweet plants were identified and owned by Shunzhao Sui from southwest University of China.

The *Arabidopsis thaliana* (Col-0) was used for plant transformation and phenotype comparison, and the tobacco (*Nicotiana benthamiana*) was planted for subcellular localization analysis. The seeds of *A. thaliana* and tobacco were stored at 4 °C, germinated on the Murashige and Skoog (MS) medium with 3% sucrose and 0.7% agar, then the plants with 4 leaves were transferred to sterile soil and cultivated in a greenhouse with a relative humidity of 70%, a photoperiod of 16-h light (120 umol m^− 2^ s^− 1^, 22 °C) /8-h dark (20 °C).

### Cloning and sequence analysis

Total RNA was extracted by Trizol reagent (Thermofisher, CN) from the wintersweet flower following the RNA extraction protocol. The cDNA was synthesized with a PrimeScript® II First Strand cDNA Synthesis Kit (TaKaRa, Japan). The cDNA sequence of *CpC3H3* was obtained from the transcriptome database of wintersweet flower [51]. The promoter sequence was validated based on the genome sequence of *Chimonanthus salicifolius* [52]. Primers for *CpC3H3*-cDNA, *CpC3H3*-CDS and promoter of *CpC3H3* amplifying were designed with primer primer 6.0 based on the transcriptome and genome sequence of *CpC3H3* (Table S1).

A BLASTX was performed to search the NCBI database for the homologues of CpC3H3 from the model plants, such as *Arabidopsis thaliana*, *Glycine max*, *Zea mays*, *Populus trichocarpa*, and several other plants like *Cinnamomum micranthum*, *Nelumbo nucifera*, *Vitis vinifera*, *Camellia sinensis*. Multiple Sequence Alignment was conducted using online MSA tool (https://www.genome.jp/tools-bin/clustalw). A neighbor-joining phylogenetic tree was constructed by using the MEGA 6.0 software with a bootstrap of 1000 replicates. Putative *cis*-acting elements were predicted by the online tool PlantCARE (http://bioinformatics.psb.ugent.be/webtools/plantcare/html/).

### Expression analysis

Total RNA extracted from various tissues was reverse-transcribed following the instructions of the primerscript RT reagent kit (Takara, Japan) to generate cDNA samples. Then quantitative real time PCR amplifications were conducted with the specific primers and cDNA template. Wintersweet *CpTublin*, *CpActin* genes, and *A. thaliana AtActin* gene were used as internal control for the expression analysis respectively. The gene expression was quantified by the comparative C_T_ method as previously used [25]. Total RNA extracted from various tissues (cotyledons, roots, stems, young leaves, mature leaves, petals, stamens and pistils) and from flower bud at floral meristem formation stage, flower organ primordium formation stages (sepal, petal, stamen, and pistil) and flowering stages [25] (Stage 1-6: bud-display-initiating bloom-bloom-early withering-late withering) were used to analyze the spatiotemporal expression of *CpC3H3* in wintersweet. Total RNA extracted from 8-week wintersweet seedlings at 0 h, 2 h, 6 h, 12 h, 24 h after treated with 20% polyethylene glycol (PEG) 6000 were used to analyze the induced expression pattern. Total RNA of twenty-day-old *A. thaliana* seedlings were used to analysis the expression pattern of the differential expression genes.

### Vector construction and plant transformation

The *CpC3H3* CDS without the stop codon was cloned into the modified vector pCAMBIA1300 which contained a 35S promoter and a GFP reporter gene to generate the plant expression vector *35S: CpC3H3-GFP*. Then the vector *35S:CpC3H3-GFP* and *35S:GFP* were transformed into the *Agrobacterium tumefaciens* strain GV3101 respectively. The *35S:CpC3H3-GFP* was then transformed to *A. thaliana* plants under the instruction of the floral dip method. Transgenic plants were selected on MS medium with 25 mg/L hygromycin to obtain homozygous transgenic plants, and then confirmed by PCR and qPCR amplifying.

### Subcellular localization

The subcellular localization of CpC3H3 was first predicted by the online tool WoLF PSORT (WoLF PSORT: Protein Subcellular Localization Prediction Tool (genscript.com)). And then roots of the 35S:CpC3H3-GFP and 35S:GFP overexpression plants were used to analyze the subcellular localization. DAPI was used to mark the location of nucleus and FM 4-64 was used to mark the position of the cell membrane. Roots of transgenic plant treated with 20% PEG 6000 for 24 h were used to analyzed the shuttling of CpC3H3. Fluorescent protein was observed by confocal microscopy (Olympus, FV-10-ASW, Japan).

### Drought tolerance assay

Transgenic *A. thaliana* T_3_ and WT seedlings were grown in pots containing a mixture of Peat: Perlite (2:1, v/v) for 4 weeks, and then 30 plants of each line were treated with 50 mL of 20% PEG6000 solution for 2 days, 50 mL of 150 mmol/L mannitol for 2 days, and planted without watering for 10 days respectively to detect the drought tolerance.

### RNA sequencing analysis

The total RNA extracted from twenty-day-old WT and T3 transgenic plants were used to construct the cDNA libraries by following the user’ instruction of TIANSeq Stranded RNA-Seq Kit (Illumina) (YAD, China). The libraries were sequenced with an Illumina Hiseq system at Beijing Genomics institution. Single-end sequences were first obtained, and adaptor sequences were removed from the raw sequences, and then the lower quality sequences (<Q20) were removed, all the reads were mapped to the reference genome TAIR 10. The differentially expressed genes (DEGs) were identified with a cut-off change more than 2 folds between WT and transgenic plants and an independent T-test *p <* 0.05. Three biological replicates of WT and overexpression line 18, 10, 14 (each line was served as a replicate of overexpression plants) were analyzed. The RNA-sequencing data set can be obtained from SRA database with an accession number of PRJNA779571.

### Determination of physiological indices

The content of proline, soluble sugar, malondialdehyde (MDA), activities of peroxidase (POD) and electrolyte leakage rates in WT and the transgenic *A. thaliana* plants those were treated with 20%PEG solution for 24 h were determined according to the method previously described [36, 53–56]. The 8th and 9th leaves of each line were collected for the determination. The osmotic potentials of the leaves of 1-year-old wintersweet, 7-days-old WT and transgenic *A. thaliana* those were treated with 20% PEG solution for 24 h were tested by following the method descripted by Vijay Paul [57].

## Supplementary Information

**Additional file 1.**

**Additional file 2.**

## Data Availability

The datasets analysed during the current study are available in the SRA (https://www.ncbi.nlm.nih.gov/sra) repository with a with an accession number of PRJNA779571.
