# Supplementary material for: A tandem CCCH type zinc finger protein gene CpC3H3 from Chimonanthus praecox promotes flowering and enhances drought tolerance in Arabidopsis
Source: BMC Plant Biol. 2022 Oct 29;22:506. doi: 10.1186/s12870-022-03877-2 (PMC9617390; doi:10.1186/s12870-022-03877-2)
Supplement: Supplementary file 1 — Additional file 1. [file 12870_2022_3877_MOESM1_ESM.docx]

Table S1 Primers used in this study

| Primer | sequence |
| --- | --- |
| CpC3H3-F | ATGCCAGATAATCGGCAGATTCAGG |
| CpC3H3-R | CTAATCAGATTGATCAGGTGGGGAGT |
| CpC3H3-q-F | AGGCGACCTCCACTTGTTTG |
| CpC3H3-q-R | CCATCTTGGTTACCCATATGAATTT |
| IPT3-q-F | CAAGCCTACCAAACATAACAAAACA |
| IPT3-q-R | TCGCTGGAGGGAAGTCTAAAGTC |
| HBI1-q-F | GATCATAAGTTGAAAGATTTCTGCAG |
| HBI1-q-R | CCCATTTAAGCCTTTCAAGTACAGA |
| ARR4-q-F | CTATTTTATTCTCATCCTCTCCTCACA |
| ARR4-q-R | TCCGACCTTCGTAAACAAGAAAC |
| ARR5-q-F | CTCTCTATCTCTCTCACGAGTCACG |
| ARR5-q-R | CGATGAACTTCCGATCAACCATAC |
| ARR7-q-F | TTTATTCTGAGTTTGACAATGGCGG |
| ARR7-q-R | GATCCACAATACTATCATCGACGGC |
| EXPA1-q-F | TTCCAATTCTAAACCAAACAACAGA |
| EXPA1-q-R | TCATTGCTCCAAGGGTAGCAATA |

Table S2 cis-element prediction of CpC3H3 promoter

| Type of cis-element | Name of cis-element | Sequence of  cis-element | Numner of cis-element | | Function |
| --- | --- | --- | --- | --- | --- |
| Light responsive elements | GATA-motif | AAGATAAGATT | 1 | | part of the light responsive element |
|  | I-box | AGATAAGG | 1 | |  |
|  | GT1-motif | GGTTAA | 2 | | light responsive |
|  | TCT-motif | TCTTAC | 1 | |  |
|  | G-box | ACACGTGT/CACGTG/CACGAC | | 3 |  |
|  | Box 4 | ATTAAT | 8 | |  |
| Hormone responsive elements | ABRE | CACGTG/ACGTG | 2 | | ABA responsive |
|  | MYC | CATTTG/CATGTG/  CAATTG | 4 | |  |
|  | TGACG-motif | TGACG | 2 | | MeJA responsive |
|  | ERE | ATTTTAAA | 1 | | Ethylene responsive |
|  | TGA-element | AACGAC | 1 | | Auxin responsive |
|  | CGTCA-motif | CGTCA | 2 | | MeJA responsive |
| Stress responsive elements | MBS | CAACTG | 1 | | Drought responsive |
|  | TC-rich repeats | GTTTTCTTAC/ATTCTCTAAC | 2 | | Defense and stress Responsive |
|  | ARE | AAACCA | 2 | | Anaerobic induction |
|  | MYB | CAACCA/TAACCA | 3 | | MYB biding site |
|  | MYB-like sequence | TAACCA | 1 | |  |
|  | CCAAT-box | CAACGG | 1 | | MYBHv1 biding site |

Table S3 123 differential expression genes

| No. | Gene ID | FoldChange | regulated | NR |
| --- | --- | --- | --- | --- |
| 1 | AT1G01190 | 12.621 | up | cytochrome P450, family 78, subfamily A, polypeptide 8 |
| 2 | AT1G02850 | 0.172 | down | beta glucosidase 11 |
| 3 | AT1G10470 | 2.685 | up | response regulator 4 |
| 4 | AT1G14890 | 3.292 | up | Plant invertase/pectin methylesterase inhibitor superfamily protein |
| 5 | AT1G17090 | 0.353 | down | transmembrane protein |
| 6 | AT1G18330 | 2.452 | up | Homeodomain-like superfamily protein |
| 7 | AT1G18400 | 2.348 | up | BR enhanced expression 1 |
| 8 | AT1G19050 | 3.904 | up | ARR7 |
| 9 | AT1G19610 | 7.586 | up | defensin-like protein |
| 10 | AT1G21520 | 0.455 | down | hypothetical protein AT1G21520 |
| 11 | AT1G21525 | 0.241 | down | -- |
| 12 | AT1G30720 | 3.313 | up | FAD-binding Berberine family protein |
| 13 | AT1G31550 | 0.444 | down | GDSL-like Lipase/Acylhydrolase superfamily protein |
| 14 | AT1G32900 | 0.222 | down | UDP-Glycosyltransferase superfamily protein |
| 15 | AT1G33730 | 0.214 | down | cytochrome P450, family 76, subfamily C, polypeptide 5 |
| 16 | AT1G43910 | 0.334 | down | P-loop containing nucleoside triphosphate hydrolases superfamily protein |
| 17 | AT1G49500 | 7.694 | up | transcription initiation factor TFIID subunit 1b-like protein |
| 18 | AT1G51270 | 2.244 | up | vesicle-associated protein 1-4 |
| 19 | AT1G56430 | 3.818 | up | nicotianamine synthase 4 |
| 20 | AT1G56650 | 0.164 | down | production of anthocyanin pigment 1 |
| 21 | AT1G57560 | 0.425 | down | myb domain protein 50 |
| 22 | AT1G62510 | 2.854 | up | Bifunctional inhibitor/lipid-transfer protein/seed storage 2S albumin superfamily protein |
| 23 | AT1G66100 | 0.398 | down | Plant thionin |
| 24 | AT1G66390 | 0.135 | down | myb domain protein 90 |
| 25 | AT1G68238 | 3.722 | up | transmembrane protein |
| 26 | AT1G68740 | 5.174 | up | EXS (ERD1/XPR1/SYG1) family protein |
| 27 | AT1G69530 | 3.069 | up | expansin A1 |
| 28 | AT1G70260 | 0.394 | down | nodulin MtN21 /EamA-like transporter family protein |
| 29 | AT1G70440 | 0.095 | down | similar to RCD one 3 |
| 30 | AT1G70560 | 2.316 | up | tryptophan aminotransferase of Arabidopsis 1 |
| 31 | AT1G74670 | 5.705 | up | Gibberellin-regulated family protein |
| 32 | AT1G74890 | 4.04 | up | response regulator 15 |
| 33 | AT1G76040 | 0.499 | down | calcium-dependent protein kinase 29 |
| 34 | AT1G78830 | 3.304 | up | Curculin-like (mannose-binding) lectin family protein |
| 35 | AT2G04050 | 0.267 | down | MATE efflux family protein |
| 36 | AT2G05540 | 4.708 | up | Glycine-rich protein family |
| 37 | AT2G15890 | 2.298 | up | maternal effect embryo arrest 14 |
| 38 | AT2G18300 | 2.329 | up | basic helix-loop-helix (bHLH) DNA-binding superfamily protein |
| 39 | AT2G18700 | 2.332 | up | trehalose phosphatase/synthase 11 |
| 40 | AT2G28660 | 2.031 | up | hypothetical protein AXX17_AT2G24760 |
| 41 | AT2G29165.1 | 0.433 | down | -- |
| 42 | AT2G30040 | 3.102 | up | mitogen-activated protein kinase kinase kinase 14 |
| 43 | AT2G30766 | 10.36 | up | hypothetical protein AT2G30766 |
| 44 | AT2G32870 | 5.999 | up | TRAF-like family protein |
| 45 | AT2G32880 | 2.739 | up | TRAF-like family protein |
| 46 | AT2G33830 | 3.746 | up | Dormancy/auxin associated family protein |
| 47 | AT2G36120 | 10.705 | up | -- |
| 48 | AT2G40610 | 2.922 | up | expansin A8 |
| 49 | AT2G41560 | 2.221 | up | autoinhibited Ca(2+)-ATPase, isoform 4 |
| 50 | AT2G43580 | 0.092 | down | Chitinase family protein |
| 51 | AT2G44080 | 0.493 | down | ARGOS-like protein |
| 52 | AT3G02040 | 4.51 | up | senescence-related gene 3 |
| 53 | AT3G13750 | 2.83 | up | beta galactosidase 1 |
| 54 | AT3G14940 | 2.242 | up | phosphoenolpyruvate carboxylase 3 |
| 55 | AT3G16770 | 2.957 | up | ethylene-responsive element binding protein |
| 56 | AT3G19030 | 3.4 | up | transcription initiation factor TFIID subunit 1b-like protein |
| 57 | AT3G21460 | 55.215 | up | Glutaredoxin family protein |
| 58 | AT3G23450 | 2.077 | up | unknown, partial |
| 59 | AT3G24982 | 0.331 | down | receptor like protein 40 |
| 60 | AT3G26200 | 0.293 | down | cytochrome P450, family 71, subfamily B, polypeptide 22 |
| 61 | AT3G26815 | 39.144 | up | -- |
| 62 | AT3G26818 | 49.856 | up | -- |
| 63 | AT3G45140 | 0.264 | down | lipoxygenase 2 |
| 64 | AT3G45160 | 4.563 | up | Putative membrane lipoprotein |
| 65 | AT3G45730 | 0.292 | down | hypothetical protein AT3G45730 |
| 66 | AT3G48100 | 3.515 | up | response regulator 5 |
| 67 | AT3G49940 | 2.985 | up | LOB domain-containing protein 38 |
| 68 | AT3G55646 | 11.892 | up | TPRXL |
| 69 | AT3G56710 | 0.407 | down | sigma factor binding protein 1 |
| 70 | AT3G57040 | 2.439 | up | response regulator 9 |
| 71 | AT3G57460 | 0.373 | down | putative protein |
| 72 | AT3G57630 | 0.493 | down | exostosin family protein |
| 73 | AT3G62930 | 22.504 | up | Thioredoxin superfamily protein |
| 74 | AT3G62950 | 5.587 | up | Thioredoxin superfamily protein |
| 75 | AT3G63110 | 5.323 | up | isopentenyltransferase 3 |
| 76 | AT4G01080 | 0.234 | down | TRICHOME BIREFRINGENCE-LIKE 26 |
| 77 | AT4G03610 | 3.557 | up | Metallo-hydrolase/oxidoreductase superfamily protein |
| 78 | AT4G15440 | 0.251 | down | hydroperoxide lyase 1 |
| 79 | AT4G15660 | 263.961 | up | Thioredoxin superfamily protein |
| 80 | AT4G15670 | 97.91 | up | At4g15670 |
| 81 | AT4G15680 | 89.752 | up | Thioredoxin superfamily protein |
| 82 | AT4G15690 | 44.063 | up | Thioredoxin superfamily protein |
| 83 | AT4G15700 | 23.852 | up | Thioredoxin superfamily protein |
| 84 | AT4G16008 | 3.585 | up | hypothetical protein AT4G16008 |
| 85 | AT4G17660 | 0.247 | down | Protein kinase superfamily protein |
| 86 | AT4G18280 | 3.094 | up | glycine-rich cell wall protein-like protein |
| 87 | AT4G23750 | 2.75 | up | cytokinin response factor 2 |
| 88 | AT4G26150 | 3.24 | up | cytokinin-responsive gata factor 1 |
| 89 | AT4G26540 | 2.025 | up | Leucine-rich repeat receptor-like protein kinase family protein |
| 90 | AT4G32340 | 3.91 | up | Tetratricopeptide repeat (TPR)-like superfamily protein |
| 91 | AT4G37540 | 3.23 | up | LOB domain-containing protein 39 |
| 92 | AT4G38340 | 0.244 | down | Plant regulator RWP-RK family protein |
| 93 | AT4G39070 | 3.295 | up | B-box zinc finger family protein |
| 94 | AT5G01600 | 0.284 | down | ferretin 1 |
| 95 | AT5G02580 | 0.276 | down | argininosuccinate lyase |
| 96 | AT5G03210 | 0.441 | down | E3 ubiquitin-protein ligase |
| 97 | AT5G03350 | 0.419 | down | Legume lectin family protein |
| 98 | AT5G04750 | 0.489 | down | F1F0-ATPase inhibitor protein |
| 99 | AT5G04770 | 4.301 | up | cationic amino acid transporter 6 |
| 100 | AT5G05250 | 6.634 | up | hypothetical protein AT5G05250 |
| 101 | AT5G05410 | 2.439 | up | DRE-binding protein 2A |
| 102 | AT5G16080 | 3.836 | up | carboxyesterase 17 |
| 103 | AT5G20150 | 2.172 | up | SPX domain-containing protein 1 |
| 104 | AT5G22380 | 0.463 | down | NAC domain containing protein 90 |
| 105 | AT5G22545 | 0.281 | down | hypothetical protein AT5G22545 |
| 106 | AT5G24200 | 0.279 | down | alpha/beta-Hydrolases superfamily protein |
| 107 | AT5G40890 | 2.912 | up | chloride channel A |
| 108 | AT5G44210 | 2.002 | up | erf domain protein 9 |
| 109 | AT5G44390 | 0.41 | down | FAD-binding Berberine family protein |
| 110 | AT5G46871 | 3.801 | up | Putative membrane lipoprotein |
| 111 | AT5G49360 | 4.481 | up | beta-xylosidase 1 |
| 112 | AT5G52320 | 0.495 | down | cytochrome P450, family 96, subfamily A, polypeptide 4 |
| 113 | AT5G52390 | 0.256 | down | PAR1 protein |
| 114 | AT5G52760 | 0.455 | down | Copper transport protein family |
| 115 | AT5G55420 | 0.391 | down | -- |
| 116 | AT5G56860 | 2.005 | up | GATA type zinc finger transcription factor family protein |
| 117 | AT5G59080 | 3.06 | up | hypothetical protein AT5G59080 |
| 118 | AT5G61660 | 3.11 | up | AT5G61660 |
| 119 | AT5G62350 | 2.512 | up | Plant invertase/pectin methylesterase inhibitor superfamily protein |
| 120 | AT5G62360 | 2.769 | up | Plant invertase/pectin methylesterase inhibitor superfamily protein |
| 121 | AT5G62920 | 15.252 | up | response regulator 6 |
| 122 | AT5G63470 | 2.213 | up | nuclear factor Y, subunit C4 |
| 123 | AT5G64800 | 0.376 | down | CLAVATA3/ESR-RELATED 21 |
