# Supplementary material for: A tandem CCCH type zinc finger protein gene CpC3H3 from Chimonanthus praecox promotes flowering and enhances drought tolerance in Arabidopsis
Source: BMC Plant Biol. 2022 Oct 29;22:506. doi: 10.1186/s12870-022-03877-2 (PMC9617390; doi:10.1186/s12870-022-03877-2)
Supplement: Supplementary file 2 — Additional file 2. [file 12870_2022_3877_MOESM2_ESM.docx]

**Supplementary Figures**


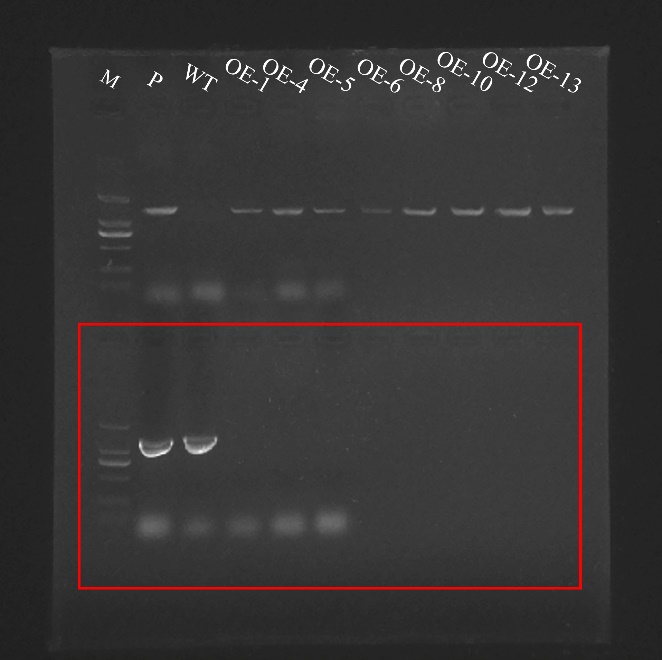

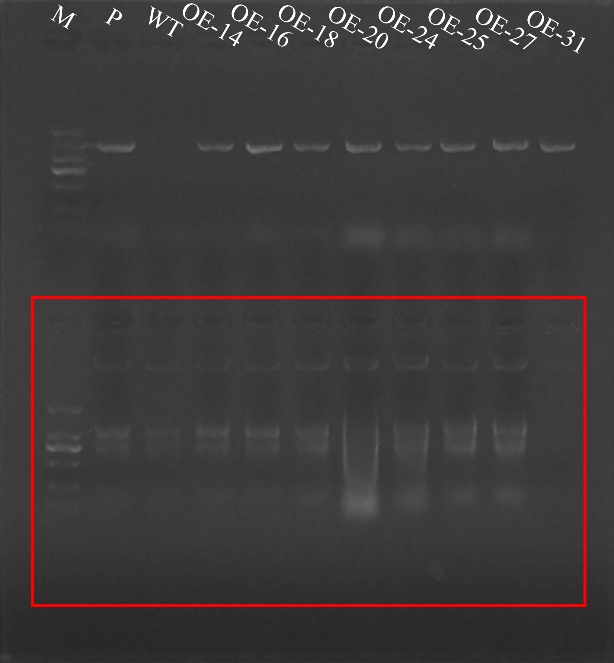


Figure S1. PCR amplication to detect the transgenic *Arabidopsis* lines.

M: marker, p: positive control, WT: Wild Type, OE1-OE31: Overexpression line 1-31. Bolts in the red box are the results of other experiments which shares no connection with this study.


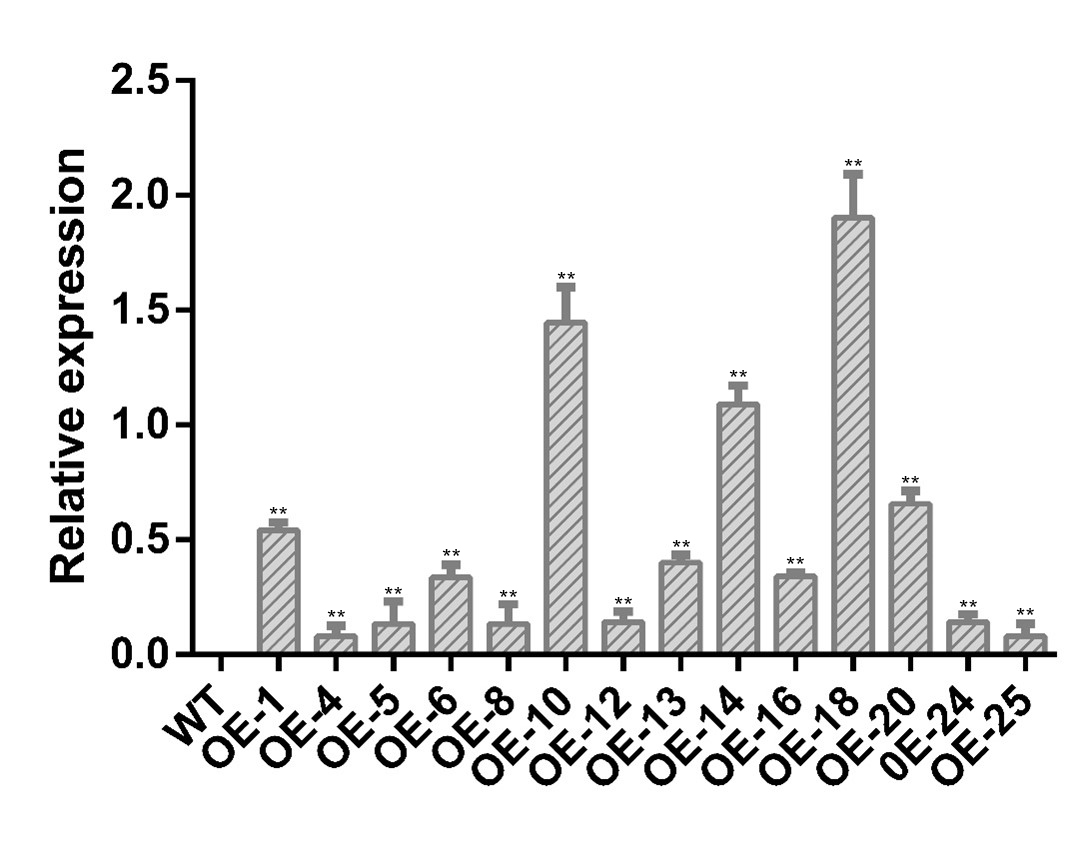


Figure S2. qRT-PCR amplication to detect the transcripts of *CpC3H3* in transgenic *Arabidopsis* lines. WT, wild type; OE1-OE25: Overexpression line 1-25; the error bars represent the standard deviation per triplicate. The ** indicate a significant difference from WT at p < 0.01which determined by the Student t-test.
